# The lipotoxicity–ferroptosis axis in the transition from insulin resistance to type 2 diabetes: mechanistic integration and therapeutic opportunities

**DOI:** 10.3389/fendo.2026.1952532

**Published:** 2026-10-01

**Authors:** Jianzhong Cui, Hongguang Li, Junying Liu, Mingjun Liu, Jinying Zhao

**Affiliations:** 1 Changchun University of Chinese Medicine, Changchun, Jilin, China; 2 The Third Affiliated Hospital of Changchun University of Chinese Medicine, Changchun, Jilin, China

**Keywords:** ferroptosis, lipotoxicity, pancreatic β cell, therapeutic target, type 2 diabetes mellitus

## Abstract

Insulin resistance is an early feature of type 2 diabetes mellitus (T2DM), but progression to persistent hyperglycemia requires failure of pancreatic β-cell compensation. Lipotoxicity develops when adipose storage and oxidative capacity are exceeded, increasing fatty-acid flux and accumulation of ceramides, diacylglycerols, and oxidizable phospholipids in liver, skeletal muscle, and pancreatic islets. Ferroptosis is an iron-dependent form of regulated cell death driven by uncontrolled phospholipid peroxidation. Lipotoxicity can increase ferroptosis susceptibility by enriching polyunsaturated fatty acid-containing membrane phospholipids, increasing organelle-derived reactive oxygen species, and weakening system Xc−–glutathione–glutathione peroxidase 4 defense; however, expansion of a redox-active iron pool is a distinct, cell- and context-dependent requirement. We organize these processes into a four-state working model spanning metabolic compensation, systemic lipid overflow and insulin resistance, ferroptosis sensitization, and β-cell decompensation. This is a non-obligate conceptual trajectory rather than a universally recognized clinical sequence. Evidence is strongest in pancreatic β-cell and liver models; direct skeletal-muscle causality is limited, and a causal role in human T2DM has not been established. Ferroptosis also has cell-specific effects: protecting β cells may be beneficial, whereas ferroptotic removal of selected inflammatory or lipid-storing cell populations can improve metabolic outcomes in some models. Upstream lipid unloading through weight loss and established metabolic treatments has the greatest clinical maturity. ACSL4/LPCAT3-dependent membrane remodeling, GPX4-centered defense, ferritinophagy, labile iron, parallel suppressor systems, and targeted delivery remain predominantly preclinical or candidate approaches. Multidimensional biomarker panels may support future mechanistic studies, but no individual marker or composite panel is currently validated to diagnose ferroptosis or a ferroptosis-susceptible phenotype in T2DM. The lipotoxicity–ferroptosis axis should therefore be viewed as a testable, tissue-specific amplifier rather than a universal or solitary cause of T2DM.

## 1 Introduction

Type 2 diabetes mellitus (T2DM) develops through the progressive interaction between impaired insulin action and inadequate pancreatic β-cell compensation (1). During early obesity, increased insulin secretion may preserve near-normal glucose concentrations despite substantial insulin resistance. This compensated state can persist for years, indicating that insulin resistance alone is insufficient to explain the transition to overt diabetes (2). Disease progression occurs when nutrient excess, ectopic lipid deposition, chronic inflammation, and organelle stress exceed the adaptive capacity of β cells and insulin-responsive tissues (3). A mechanistic framework for this transition must therefore explain how a potentially reversible metabolic disturbance becomes sustained cellular dysfunction and loss of functional β-cell mass (4).

Lipotoxicity describes cellular injury caused by an imbalance among lipid uptake, synthesis, storage, oxidation, and export, rather than by lipid abundance per se (5, 6). When the safe storage capacity of adipose tissue is exceeded, non-esterified fatty acids and bioactive lipid intermediates accumulate in the liver, skeletal muscle, pancreatic islets, and other non-adipose tissues (7, 8). Diacylglycerols and ceramides impair insulin signaling, whereas saturated fatty acids promote endoplasmic reticulum stress, mitochondrial dysfunction, inflammatory signaling, and maladaptive membrane remodeling (8–10). Neutral triglycerides and lipid droplets may initially buffer excess fatty acids, but this protection becomes insufficient during sustained glucolipotoxic exposure (11–13).

Ferroptosis is a regulated form of cell death driven by iron-dependent phospholipid peroxidation (14, 15). It is controlled by the availability of oxidizable phospholipid substrates, the size and localization of the labile iron pool, and the capacity of glutathione peroxidase 4 (GPX4)-dependent and parallel antioxidant systems to terminate lipid radical chain reactions (15–17). Recent methodological recommendations emphasize that increased reactive oxygen species (ROS), malondialdehyde, iron, or reduced GPX4 expression alone does not establish ferroptosis (18). Convincing evidence requires a coherent combination of oxidized phospholipid accumulation, iron dependence, rescue by mechanistically distinct ferroptosis inhibitors, genetic perturbation, and exclusion of alternative death programs.

Lipotoxicity can supply or amplify two major components of ferroptosis susceptibility: oxidizable membrane lipids and sustained oxidative pressure, while also weakening lipid-peroxide defense (19–21). It does not, however, invariably expand the redox-active iron pool in every affected cell; iron uptake, storage, export, ferritinophagy, and organelle localization remain separately regulated and tissue specific. When these lipid/redox changes converge with permissive iron handling, ferroptosis may convert metabolic stress into persistent loss of insulin-producing or insulin-responsive cells (19, 22, 23). We therefore propose the lipotoxicity–ferroptosis axis as a context-dependent amplifier linking adipose lipid overflow and multiorgan insulin resistance to β-cell decompensation. This review integrates evidence across adipose tissue, liver, skeletal muscle, and pancreatic islets; distinguishes direct causal data from associative evidence; evaluates therapeutic targets according to translational maturity; and defines the experimental and clinical requirements needed to test this framework.

## 2 Literature search and evidence selection

This narrative review used PubMed as the primary database and focused on literature published from January 2003 to 25 August 2026. Search terms included combinations of “ferroptosis,” “lipotoxicity,” “glucolipotoxicity,” “insulin resistance,” “type 2 diabetes,” “pancreatic beta cell,” “GPX4,” “SLC7A11,” “ACSL4,” “FSP1,” “iron metabolism,” “lipid peroxidation,” “biomarker,” “exosome,” and “targeted delivery.” We prioritized original studies that directly examined T2DM, insulin resistance, pancreatic β cells, or metabolically active tissues. Recent high-quality reviews and methodological consensus articles were included to define ferroptosis and place organ-specific findings in context. Retracted articles, expressions of concern, duplicate preprints, and studies without sufficient methodological information were excluded. Because this was a narrative rather than systematic review, no formal risk-of-bias score or meta-analysis was performed. Evidence was organized by biological proximity to the proposed axis: direct genetic or pharmacological causality, rescue experiments, multilevel cellular or animal evidence, human tissue or omics association, and mechanistic extrapolation.

## 3 Conceptual boundaries: lipid overload, lipotoxicity, and ferroptosis susceptibility

Fatty acids are fuels, structural components of membranes, and signaling molecules. Their biological effects depend on chain length, saturation, intracellular localization, and the capacity of a cell to esterify or oxidize them (24–26). Palmitate promotes ceramide synthesis, membrane rigidity, inflammatory signaling, and endoplasmic reticulum stress, whereas mono-unsaturated fatty acids can redirect saturated fatty acids toward triglyceride storage and reduce toxicity (27–29). Diacylglycerol species can activate protein kinase C isoforms and disrupt insulin receptor signaling (30). Free cholesterol and oxidized sterols perturb membranes and mitochondria. Conversely, triglyceride synthesis and lipid-droplet formation may be protective when they isolate fatty acids from signaling and oxidative pathways (31).

This distinction is essential for ferroptosis. The quantity of total cellular lipid does not directly predict susceptibility; the composition and subcellular distribution of phospholipids are more informative. Human EndoC-βH1 cells show fatty-acid-structure-dependent amplification of lipotoxicity by glucose, while human islets exposed to glucolipotoxicity release pyruvate and citrate, suggesting that metabolic adaptation can precede overt cell death (32). Lipid droplets, desaturation, and the glycerolipid/non-esterified fatty-acid cycle can preserve stimulus–secretion coupling during early nutrient excess (33). Ferroptosis becomes more plausible when these buffering pathways fail and polyunsaturated fatty acid-containing phospholipids, redox-active iron, and insufficient peroxide detoxification converge in the same membrane compartment (17).

For organizational purposes, we define three working states: lipid loading, lipotoxic stress, and ferroptosis commitment. These terms are not presented as universally recognized clinical stages and need not occur in a fixed sequence. Lipid loading may remain adaptive; lipotoxic stress denotes impaired signaling or organelle function that can still be reversible; and ferroptosis commitment is considered only when iron-dependent membrane lipid peroxidation becomes self-propagating and cannot be contained by GPX4 or parallel defense systems. Depending on tissue, genotype, iron handling, and exposure history, ferroptosis susceptibility may arise concurrently with, follow, or remain partly independent of selected metabolic abnormalities. This non-obligate framework avoids treating every lipid-induced increase in ROS as ferroptosis and provides testable intervention windows rather than a universal natural history.

## 4 The multiorgan relay from lipid overflow to insulin resistance

### 4.1 Adipose tissue: failure of safe storage and context-dependent ferroptosis

Healthy white adipose tissue protects other organs by expanding through adipocyte hyperplasia, storing fatty acids in lipid droplets, and suppressing lipolysis in response to insulin (34). In obesity, adipocyte hypertrophy, hypoxia, extracellular matrix remodeling, and immune-cell infiltration impair this buffering function (35). Increased circulating fatty-acid flux and altered adipokines or extracellular vesicles transmit stress to the liver, skeletal muscle, and pancreatic islets (36). These events establish systemic lipotoxicity before overt hyperglycemia appears.

The role of ferroptosis within adipose tissue is not uniformly detrimental. Gpx4 deletion-mediated ferroptosis in macrophages alleviated obesity-associated insulin resistance in mice, consistent with removal of a pathogenic inflammatory population (37). In a separate study, non-lethal activation of adipocyte ferroptotic signaling through ACSL4 or ferritin perturbation reduced lipid accumulation and mitigated diet-induced metabolic dysfunction, although this does not establish the safety of inducing adipocyte death in humans (38). Conversely, ferroptotic injury to vascular endothelium can be harmful, and effects on adipose progenitors, mature adipocytes, and immune-cell subsets may differ (39–41). These observations make adipose ferroptosis a double-edged, cell-state-dependent process rather than a uniformly protective or pathogenic pathway. Systemic ferroptosis inhibition could protect β cells while preserving harmful immune cells, whereas systemic induction could damage vascular or regenerative compartments. Therapeutic strategies must therefore consider cell identity, dose, whether signaling is sublethal or lethal, and disease stage.

### 4.2 Liver: diacylglycerol signaling, steatosis, and iron-sensitive oxidative injury

The liver receives fatty acids released from adipose tissue and synthesizes additional lipid through *de novo* lipogenesis. Accumulation of specific diacylglycerols activates protein kinase Cϵ and impairs insulin receptor signaling, reducing insulin-mediated suppression of gluconeogenesis (30). Excess fatty-acid oxidation increases electron leakage and ROS production, while endoplasmic reticulum stress and inflammatory signaling further disrupt insulin action (42, 43). This combination contributes to metabolic dysfunction-associated steatotic liver disease (MASLD), which feeds back to systemic insulin resistance through increased glucose production, altered lipoprotein secretion, and hepatokine signaling.

Iron overload or altered intracellular iron handling can shift a steatotic hepatocyte towards ferroptosis. Proposed mechanisms include suppression of the JAK2/STAT3/SLC7A11 axis, insufficient NRF2-mediated defense, increased ferritinophagy, and enrichment of oxidizable phospholipids (43–45). Experimental iron-dextran and ferric-ammonium-citrate models demonstrate that iron overload can produce hepatocyte lipid peroxidation and insulin resistance even when the initiating exposure is independent of adipose lipid overflow; chelation partially reverses these changes (45). Such models show that hepatocytes are capable of iron-driven ferroptotic injury, but they do not by themselves prove that ferroptosis is a common upstream cause of human T2DM. Studies of diabetes-associated liver injury and arsenic-induced hepatic insulin resistance further support relationships among disturbed iron metabolism, arachidonic-acid remodeling, ferroptosis-associated readouts, and impaired insulin signaling (46, 47). Much of this evidence remains derived from toxicant, iron-loading, high-fat-diet, or combined high-fat-diet/streptozotocin models, and direct demonstration that hepatocyte ferroptosis precedes and causes human hepatic insulin resistance is limited. The apparently stronger iron dependence in liver probably reflects its central roles in systemic iron sensing, hepcidin regulation, ferritin storage, first-pass fatty-acid exposure, and reticuloendothelial iron exchange. Other tissues are not “iron independent”; rather, redox-active iron, its subcellular localization, and ferroptosis-specific rescue have been measured less consistently outside liver and β-cell models.

### 4.3 Skeletal muscle: lipid quality and organelle mismatch

Skeletal muscle is responsible for most postprandial insulin-stimulated glucose disposal. Intramyocellular triglyceride content does not invariably indicate insulin resistance, as endurance-trained individuals can maintain high lipid stores and normal insulin sensitivity. The pathogenic variables are lipid-droplet turnover, mitochondrial oxidative matching, and the abundance and localization of ceramides and diacylglycerols. In obesity, these lipid species, together with inflammation and oxidative stress, inhibit IRS1–PI3K–AKT signaling and GLUT4 translocation (48, 49).

Direct evidence that skeletal-muscle ferroptosis drives progression to T2DM is weaker than that for pancreatic β cells or liver (50). Ferroptosis is iron-dependent by definition in every tissue. The current gap in muscle is not evidence for an iron-independent form, but the scarcity of studies that directly quantify labile or compartmentalized iron and demonstrate rescue by mechanistically distinct ferroptosis inhibitors or genetic perturbation in metabolic muscle models (51). Muscle oxidative stress and membrane lipid remodeling therefore indicate possible susceptibility but are not sufficient to establish ferroptotic injury.

### 4.4 Inter-organ communication and feed-forward amplification

The metabolic organs described above do not progress independently. Adipose lipid spillover increases hepatic lipid uptake and muscle exposure, while hepatic very-low-density lipoprotein secretion redistributes triglyceride-derived fatty acids to peripheral tissues (52, 53). Insulin-resistant liver and muscle increase the secretory demand placed on β cells. In parallel, adipokines, hepatokines, myokines, inflammatory cytokines, and extracellular vesicles alter cellular redox programs and lipid handling in distant organs (54, 55). The result is a feed-forward network in which insulin resistance increases compensatory insulin secretion, hyperinsulinemia can stimulate lipogenesis, and continued lipid excess further raises secretory and oxidative stress in β cells.

A multiorgan interpretation also clarifies why a treatment can improve glucose homeostasis without directly inhibiting ferroptosis in every tissue. Weight loss can reduce liver and intermuscular fat and improve insulin sensitivity across several organs, thereby lowering β-cell secretory demand (56, 57). These established metabolic benefits should not be attributed to ferroptosis modulation without pathway-specific evidence. Conversely, β-cell-directed inhibition of ferroptotic stress can preserve insulin secretion in cellular and mouse models while leaving upstream insulin resistance incompletely corrected (58). Optimal disease modification will probably require upstream metabolic intervention combined, when supported by future mechanistic biomarkers, with tissue-selective protection of vulnerable cells.

## 5 Molecular convergence of lipotoxicity and ferroptosis

### 5.1 Oxidizable phospholipid substrates: the ACSL4–LPCAT3 axis

Ferroptosis is executed through peroxidation of membrane phospholipids containing polyunsaturated fatty acids (PUFAs), rather than through nonspecific oxidation of all cellular lipids (59). Acyl-CoA synthetase long-chain family member 4 (ACSL4) activates arachidonic and adrenic acids, and lysophosphatidylcholine acyltransferase 3 (LPCAT3) incorporates these acyl chains into phospholipids (60, 61). Lipoxygenases, cytochrome P450 oxidoreductase, mitochondrial or NADPH oxidase-derived radicals, and non-enzymatic chain reactions then promote phospholipid hydroperoxide accumulation (62).

Palmitate is not itself a canonical peroxidizable PUFA substrate, but it can increase ferroptosis susceptibility by stimulating ceramide signaling, increasing desaturase demand, perturbing organelles, and remodeling membrane composition (63, 64). Experimental work directly links palmitate-induced ceramide signaling to β-cell ferroptosis (63). The apparent paradox is therefore resolved by separating the initiating lipid from the final peroxidized phospholipid: saturated fatty acids create a cellular state in which PUFA-phospholipid oxidation becomes more difficult to control.

### 5.2 Lipid peroxide defense: system Xc−–GSH–GPX4 and parallel pathways

The cystine/glutamate antiporter system Xc−, composed of SLC7A11 and SLC3A2, imports cystine in exchange for intracellular glutamate. Cystine reduction supports glutathione (GSH) synthesis, and GPX4 uses GSH to reduce phospholipid hydroperoxides to non-toxic alcohols. High glucose and lipotoxic stress can weaken this pathway through NADPH depletion, altered glutamate metabolism, CHAC1-mediated GSH degradation, transcriptional repression or ubiquitination of SLC7A11, and reduced GPX4 abundance or activity (65). β cells may be particularly vulnerable because their antioxidant reserve is relatively limited and their secretory function imposes a high oxidative and endoplasmic reticulum burden.

Ferroptosis suppressor protein 1 (FSP1) regenerates reduced coenzyme Q at the plasma membrane; dihydroorotate dehydrogenase (DHODH) supports mitochondrial coenzyme Q reduction; and GTP cyclohydrolase 1 (GCH1)-tetrahydrobiopterin provides another radical-trapping system (66, 67). These GPX4-parallel pathways are established components of general ferroptosis biology and help explain why equivalent reductions in GPX4 do not produce the same outcome in every cell. In T2DM, however, direct tissue-specific causal evidence, dose–response data, and *in vivo* rescue studies remain sparse. FSP1, DHODH, and GCH1/BH4 should therefore be regarded as mechanistic candidates rather than confirmed diabetes therapeutic targets.

### 5.3 Labile iron and ferritinophagy

Ferroptosis depends on the redox-active ferrous iron pool, not simply on total tissue iron or serum ferritin. Increased transferrin receptor-dependent uptake, reduced ferroportin-mediated export, heme degradation, lysosomal iron release, and nuclear receptor coactivator 4 (NCOA4)-mediated ferritinophagy can enlarge the labile iron pool and accelerate Fenton chemistry. Lysosomes are therefore both iron-processing organelles and critical hubs connecting autophagy to ferroptosis (68, 69).

This distinction is clinically important because ferritin is also an acute-phase reactant. T2DM, MASLD, obesity, and chronic inflammation can increase circulating ferritin without demonstrating intracellular ferroptosis (70). Tissue interpretation should combine transferrin saturation, labile iron or iron imaging, oxidized phospholipids, and spatial localization. Iron chelation based solely on elevated ferritin could cause iron deficiency, impair mitochondrial function, and reduce exercise capacity.

### 5.4 Organelle stress and regulatory feedback

Excess fatty-acid oxidation increases mitochondrial electron leakage and consumes NADPH. Endoplasmic reticulum stress activates PERK–eIF2α–ATF4 and IRE1–JNK signaling, altering amino-acid and lipid metabolism (71). Lysosomal dysfunction and excessive ferritinophagy release iron, while damaged mitochondria and membranes generate additional lipid radicals (68). These events create a positive-feedback loop in which organelle dysfunction increases lipid peroxidation and lipid peroxidation further damages organelles.

Epitranscriptomic and post-translational regulation adds further context dependence. N6-methyladenosine readers, deubiquitination, microRNAs, and transcription factors such as NFAT5, NRF2, and BACH1 regulate SLC7A11, GPX4, PRDX2, CHAC1, and related genes. Ferroptosis is consequently not an isolated terminal pathway but an integrated phenotype emerging from lipid metabolism, redox control, autophagy, inflammation, and organelle quality control.

### 5.5 A threshold model of the lipotoxicity–ferroptosis axis

We propose that ferroptosis susceptibility is determined by the balance between pro-ferroptotic load and protective capacity. Pro-ferroptotic components include PUFA-phospholipid abundance, lipid-radical initiation, redox-active iron, and organelle-derived ROS; protective components include GPX4–GSH activity, FSP1–coenzyme Q, DHODH, GCH1–tetrahydrobiopterin, lipid-droplet buffering, and membrane repair. Lipotoxicity commonly increases lipid substrate and oxidative pressure and may erode peroxide defense, but it need not expand redox-active iron. Iron handling must therefore be assessed as an independent, cell-specific axis. “Threshold” is used here as a conceptual commitment boundary, not a measured universal value. When lipid/redox susceptibility and permissive iron handling converge, ferroptosis may amplify metabolic dysfunction. The balance is likely cell- and context-specific: susceptibility may be high in stressed β cells, variable in hepatocytes, incompletely defined in skeletal muscle, and potentially beneficial when selected inflammatory or lipid-storing cell populations are targeted. **Figure 1** summarizes this non-obligate convergence, and **Table 1** compares the evidence and causal gaps across tissues.

**Figure 1:**
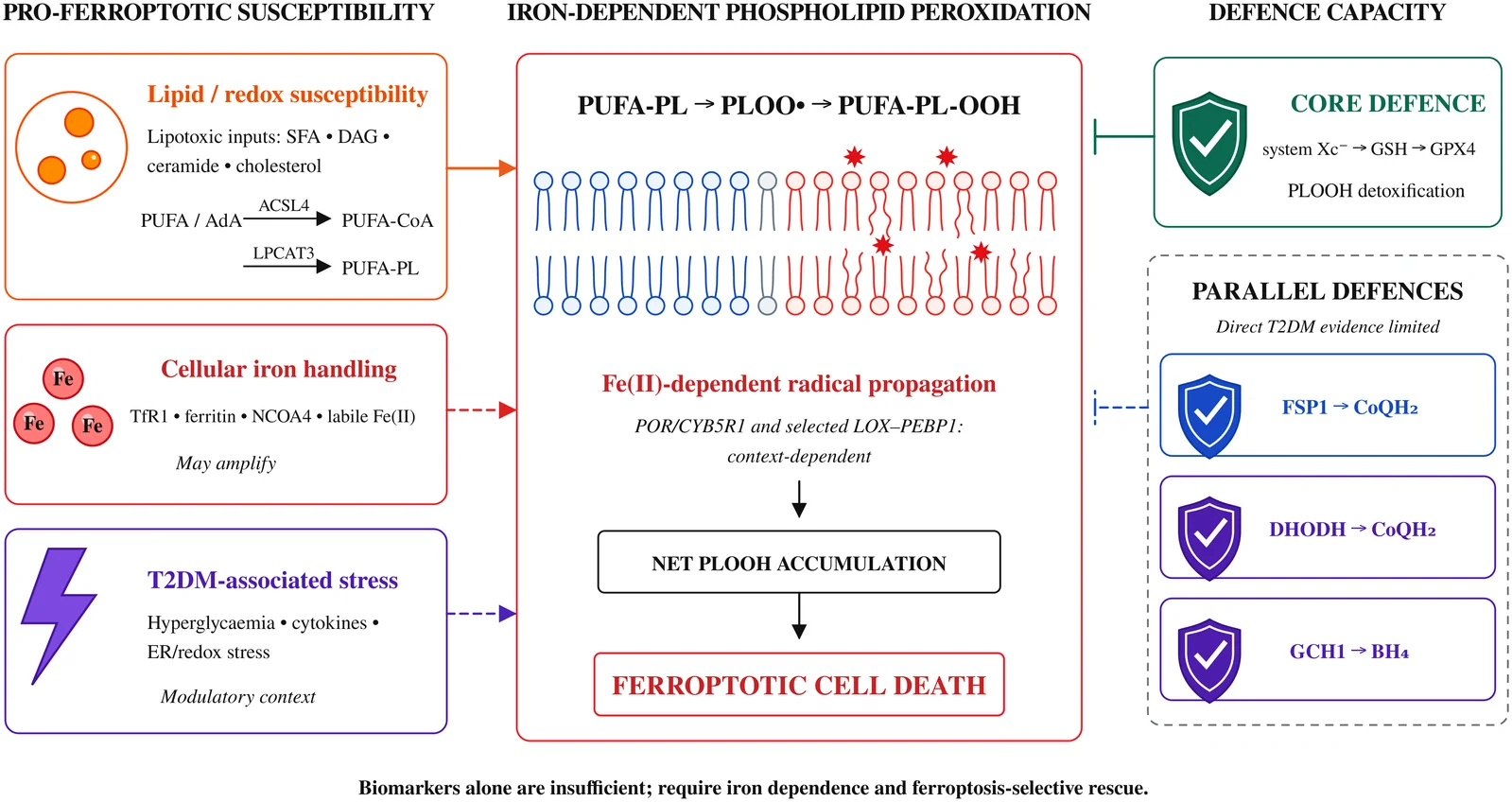
Non-obligate molecular convergence of lipotoxicity and ferroptosis. Lipid/redox susceptibility and cell-specific iron dysregulation are shown as separable inputs that may converge on iron-dependent phospholipid peroxidation. ACSL4 activates PUFA substrates, including adrenic-acid species, to PUFA-CoA, which LPCAT3 incorporates into membrane PUFA-PL. Lipotoxicity does not necessarily expand the labile iron pool in every tissue. The system Xc−–GSH–GPX4 axis is shown as the core defense, whereas FSP1–CoQH2, DHODH–CoQH2, and GCH1–BH4 are parallel candidate systems with limited direct T2DM evidence. Individual lipid-peroxidation markers or changes in GPX4, ACSL4, and Fe(II) are supportive readouts; assignment of ferroptosis requires iron dependence and mechanistically selective rescue. AdA, adrenic acid; ACSL4, acyl-CoA synthetase long-chain family member 4; BH4, tetrahydrobiopterin; CoQH2, ubiquinol; CYB5R1, cytochrome b5 reductase 1; DAG, diacylglycerol; DHODH, dihydroorotate dehydrogenase; FSP1, ferroptosis suppressor protein 1; GCH1, GTP cyclohydrolase 1; GSH, glutathione; LOX, lipoxygenase; NCOA4, nuclear receptor coactivator 4; PEBP1, phosphatidylethanolamine-binding protein 1; PLOO•, phospholipid peroxyl radical; PLOOH, phospholipid hydroperoxide; POR, cytochrome P450 oxidoreductase; PUFA-PL, polyunsaturated fatty-acid-containing phospholipid; SFA, saturated fatty acid; TfR1, transferrin receptor 1. Original conceptual schematic constructed and manually refined in Adobe Illustrator (Adobe Inc.) using vector-design and AI-assisted functions; no published image was reproduced or adapted.

**Table 1:** Tissue- and cell-specific evidence for the lipotoxicity–ferroptosis axis in progression from insulin resistance to T2DM.

Tissue/cell population	Lipotoxic/metabolic context	Iron-dependent ferroptosis evidence	Direction of effect on IR-to-T2DM progression	Evidence strength and principal causal gap
Adipocytes	Storage overload, hypertrophy, impaired lipid buffering	ACSL4/ferritin perturbation and non-lethal ferroptotic signaling affected adiposity in mice (38, 40, 41); lethal injury and human relevance remain uncertain	May reduce lipid storage in selected models, but injury to mature or progenitor cells could worsen spillover	Model-specific experimental evidence; cell-state, dose, and human safety unresolved
Adipose-tissue macrophages	Inflammation and obesity-associated immune activation	Macrophage Gpx4 deletion caused ferroptosis and improved insulin resistance in mice (37)	Potentially beneficial removal of a pathogenic inflammatory population	Direct mouse genetic evidence; generalizability to other macrophage subsets and humans unknown
Hepatocytes	DAG–PKCϵ signaling, fatty-acid oxidation overload, MASLD	Labile iron, SLC7A11/GPX4 suppression, lipid peroxidation, and chelator rescue in iron-loading and metabolic models (45–47)	Can worsen hepatic insulin resistance, gluconeogenesis, and liver injury	Supportive-to-direct model evidence; iron-loading and toxicant models do not establish common human T2DM causality
Skeletal myocytes	Ceramides, DAGs, lipid-droplet–mitochondrial mismatch	Oxidative and membrane-remodeling evidence, but labile iron, iron dependence, and specific rescue are seldom demonstrated (50, 51)	Possible contribution to impaired GLUT4 translocation and glucose disposal	Strong metabolic association; weak ferroptosis-specific causality
Pancreatic β cells	Palmitate/glucolipotoxicity, ER, mitochondrial, and lysosomal stress	ACSL4 remodeling, GPX4/PRDX2 defense, iron stress, and pharmacological and genetic rescue in cell and mouse models (20, 58, 63)	Impaired secretion, loss of identity, and reduced functional β-cell mass	Relatively strong cellular/mouse causality; human evidence remains associative
Islet macrophages and endothelium	Local inflammation, oxidized lipids, extracellular-vesicle communication	Altered lipid peroxidation and iron handling are plausible, but direct T2DM rescue evidence is sparse (39, 75)	Context-dependent inflammatory or microvascular effects	Preliminary/mechanistic extrapolation

ACSL4, acyl-CoA synthetase long-chain family member 4; DAG, diacylglycerol; GPX4, glutathione peroxidase 4; IR, insulin resistance; MASLD, metabolic dysfunction-associated steatotic liver disease; PRDX2, peroxiredoxin 2; T2DM, type 2 diabetes mellitus.

Ferroptosis is iron-dependent by definition in all tissues. “Limited iron-handling evidence” means that redox-active iron and ferroptosis-specific rescue have not been directly demonstrated in the specified metabolic model; it does not imply iron-independent ferroptosis. Evidence terms distinguish direct causal experimental evidence, supportive experimental evidence, human association, and mechanistic extrapolation.

### 5.6 Crosstalk with insulin signaling and inflammation

The relationship between ferroptosis and insulin resistance is likely bidirectional. Impaired insulin signaling increases adipose lipolysis and hepatic glucose production, thereby raising lipid and glucose exposure in other tissues. Hyperglycemia increases mitochondrial ROS, protein glycation, and NADPH demand, while lipotoxicity perturbs membrane composition and organelle function. These changes lower the ferroptosis threshold (24, 72). In the opposite direction, lipid peroxidation products can modify insulin receptor substrates, mitochondrial enzymes, ion channels, and cytoskeletal proteins, potentially worsening insulin action before cell death occurs (73). Thus, ferroptosis susceptibility may impair metabolic function even when the number of ferroptotically dying cells is small.

Inflammation adds another layer. Cytokines can alter iron sequestration, ferritin expression, hepcidin signaling, cystine metabolism, and NRF2 activity (74). Activated macrophages generate ROS and can deliver oxidized lipids to neighboring cells (75). Ferroptotic parenchymal cells may release oxidized phospholipids and damage signals that recruit or reprogram immune cells (76). However, the immunological outcome depends on the tissue, stage, and clearance of dying cells. Rapid removal of ferroptotic inflammatory cells may resolve inflammation, whereas persistent ferroptotic injury to hepatocytes, endothelium, or β cells may sustain it.

These interactions caution against a linear model in which insulin resistance simply causes ferroptosis and ferroptosis simply causes diabetes. A more realistic view is a nonlinear feedback system with tissue-specific thresholds. Early metabolic changes may be reversible and dominated by signaling defects; later stages may include irreversible loss of functional cell mass. This distinction matters for endpoints: improving insulin signaling within days does not prove prevention of ferroptosis, while preserving cell survival without reducing insulin resistance may not normalize glycemia.

## 6 Pancreatic β-cell decompensation: the gate from insulin resistance to T2DM

During early nutrient excess, β cells increase insulin biosynthesis and secretion. Lipid droplets, the glycerolipid/non-esterified-fatty-acid cycle, and adaptive autophagy can temporarily buffer fatty acids and support stimulus–secretion coupling (33). Chronic combined hyperglycemia and lipid excess eventually produces endoplasmic reticulum, Golgi, mitochondrial, and lysosomal stress; defective proinsulin processing; impaired granule formation; and loss of mature β-cell identity. Apoptosis, pyroptosis, excessive autophagy, dedifferentiation, and ferroptosis may coexist rather than occur as mutually exclusive alternatives.

The β-cell evidence for ferroptosis is increasingly coherent but remains model dependent. Palmitate or high-glucose exposure increases lipid-peroxidation-associated readouts, ACSL4 activity, iron stress, or ferroptosis susceptibility in β-cell lines and animal islets (63). Metformin (20), silibinin (77), berberine (78), grape-seed procyanidins (79), and targeted molecular interventions can restore GPX4- or NRF2-related defense, improve mitophagy, or reduce ferroptosis-associated injury. β-cell-specific NFAT5 suppression in an obese T2DM mouse model reduced ferroptotic injury and improved insulin secretion, providing stronger cell-selective experimental causality (58). Human-islet transcriptomic and single-cell analyses associate ferroptosis-related genes with impaired β-cell function or dedifferentiation (80). These observations support biological plausibility and causality in specific experimental systems, but they do not establish ferroptosis as the dominant cause of human β-cell failure.

Important limitations remain. Many studies use INS-1, MIN6, or NIT-1 cells, supraphysiological palmitate concentrations, or high-fat-diet/streptozotocin models that combine obesity with direct β-cell toxicity and insulin deficiency (22, 63). Some define ferroptosis using only ROS, malondialdehyde, Fe2+, GPX4, or ACSL4 measurements, without genetic manipulation or rescue by mechanistically distinct inhibitors (22, 63). The field therefore needs experiments in human primary islets, stem-cell-derived islets, and multicellular models that reproduce chronic patient-level fatty-acid and glucose fluctuations.

We summarize the evidence using four working states rather than universally recognized clinical stages. State 1 is reversible compensation, characterized by adipose expansion and increased β-cell secretion. State 2 is systemic metabolic dysfunction, in which lipid overflow can contribute to hepatic and skeletal-muscle insulin resistance. State 3 is ferroptosis sensitization, defined by convergence of oxidizable membrane lipids, permissive cell-specific iron handling, and declining antioxidant reserve in β cells or selected tissues. State 4 is decompensation, when dedifferentiation and cell death exceed regenerative and secretory adaptation, producing persistent hyperglycemia. These states are non-obligate and may overlap, occur in a different order, or be bypassed. Ferroptosis susceptibility may accompany, follow, or remain partly independent of particular metabolic abnormalities. In this model, ferroptosis is a possible accelerator of transition towards β-cell decompensation, not the sole initiating event or an inevitable step in T2DM. **Figure 2** summarizes this context-dependent trajectory.

**Figure 2:**
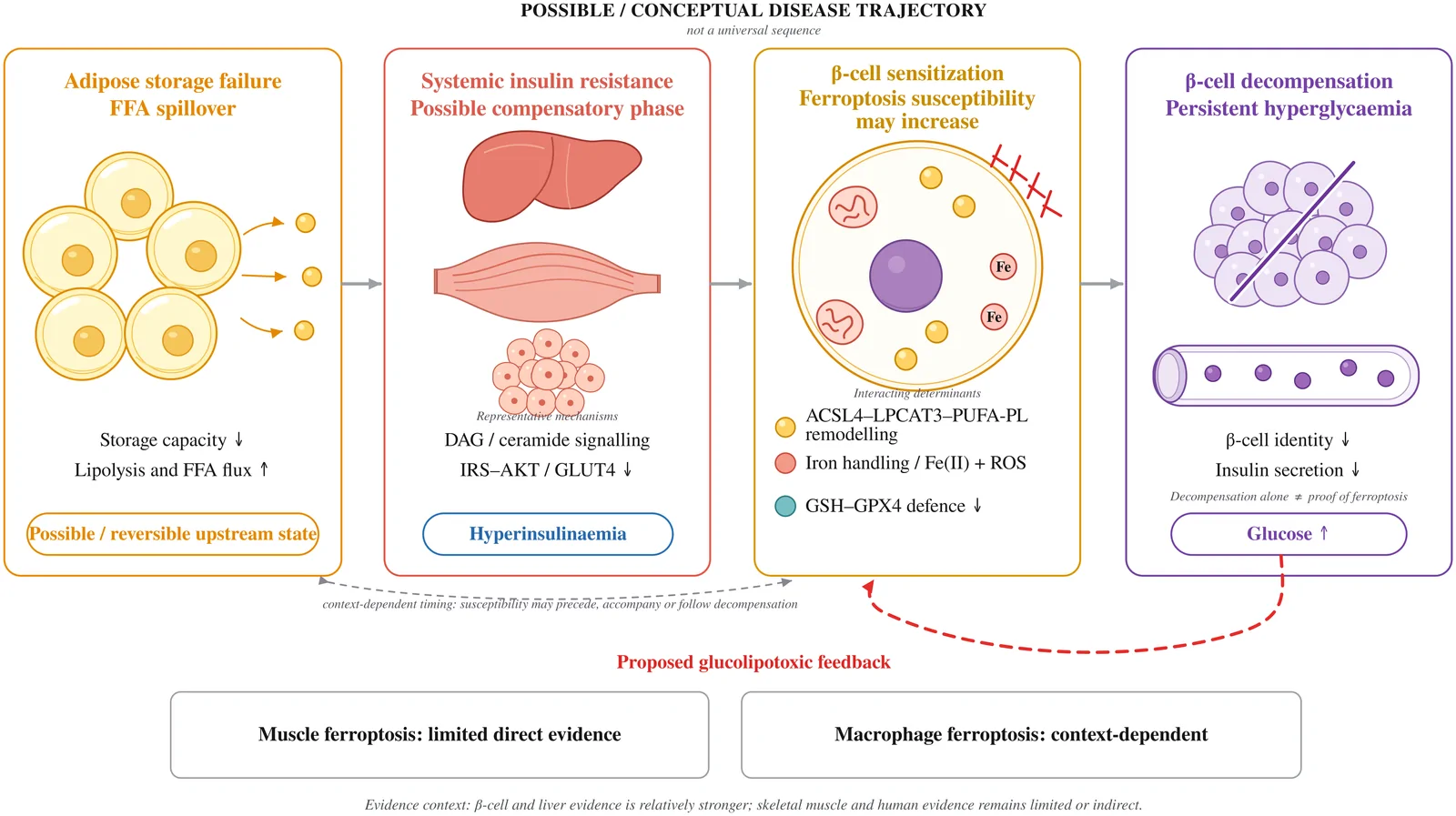
Context-dependent working model of progression from insulin resistance to T2DM. The panels show a possible, non-universal trajectory from adipose storage failure and systemic insulin resistance to β-cell ferroptosis sensitization and decompensation. These states may overlap, occur in a different order, or be bypassed; susceptibility may precede, accompany, or follow decompensation. Lipid remodeling, cell-specific iron handling, Fe(II), reactive oxygen species, and declining GSH–GPX4 defense are interacting determinants rather than a validated clinical sequence. Persistent hyperglycemia and glucolipotoxicity are shown as a proposed feedback loop. The image explicitly identifies limited direct skeletal-muscle and human evidence and context-dependent macrophage effects; β-cell decompensation alone is not proof of ferroptosis. ACSL4, acyl-CoA synthetase long-chain family member 4; DAG, diacylglycerol; FFA, free fatty acid; GLUT4, glucose transporter type 4; GPX4, glutathione peroxidase 4; GSH, glutathione; IRS, insulin receptor substrate; LPCAT3, lysophosphatidylcholine acyltransferase 3; PUFA-PL, polyunsaturated fatty-acid-containing phospholipid. Original conceptual schematic constructed and manually refined in Adobe Illustrator (Adobe Inc.) using vector-design and AI-assisted functions; no published image was reproduced or adapted.

## 7 Therapeutic opportunities

### 7.1 Reduce upstream lipid overflow

The most clinically mature strategy is to reduce the upstream lipid burden through negative energy balance, physical activity, weight loss, improved adipose insulin sensitivity, and suppression of excessive hepatic lipogenesis (81). These interventions simultaneously reduce fatty-acid flux, ceramides, diacylglycerols, inflammation, and oxidative pressure, rather than blocking only the terminal death pathway. Peroxisome proliferator-activated receptors, diacylglycerol acyltransferase 1, lipid-droplet–mitochondrial coupling, CD36-mediated uptake, and stearoyl-CoA desaturase are candidate lipid-partitioning nodes (82). Their effects are tissue-specific: enhanced neutral-lipid storage may restore buffering in adipose tissue but worsen steatosis if sustained in liver.

### 7.2 Restore GPX4-dependent and parallel defense

Increasing cysteine availability, GSH synthesis, or GPX4 activity is the most frequently studied downstream approach. γ-Glutamylcysteine, NRF2-activating compounds, hydrogen sulfide-related metabolites, vitamins, and polyphenols can increase reducing capacity and attenuate ferroptosis in preclinical models (83, 84). Long-term systemic NRF2 activation may alter drug metabolism, immune function, and tumor surveillance, while indiscriminate GPX4 enhancement could preserve metabolically harmful inflammatory cells. FSP1, DHODH, and GCH1 represent attractive complementary targets, but direct evidence in T2DM is insufficient for clinical claims.

### 7.3 Limit redox-active iron and phospholipid radical propagation

Iron chelation, inhibition of ferritinophagy, reduction of ACSL4–LPCAT3-dependent PUFA-phospholipid loading, and radical-trapping antioxidants can suppress ferroptosis in experimental systems (68). Ferrostatin-1 and liproxstatin-1 are valuable mechanistic tools but are not approved diabetes therapies; pharmacokinetics, tissue exposure, and long-term safety remain limiting (22). Clinically approved chelators should not be extrapolated to people with T2DM without demonstrated iron overload. A more plausible future strategy is short-duration, biomarker-guided, tissue-targeted modulation of labile iron or membrane lipid composition (85).

### 7.4 Reposition existing glucose-lowering therapies

Metformin suppresses β-cell ferroptosis-associated injury in several cellular and animal models through pathways involving GPX4, ACSL4, AMP-activated protein kinase, and mitochondrial homeostasis. Liraglutide reduces lipid load and ferroptosis-associated signaling through AMPK/acetyl-CoA carboxylase in T2DM-associated fatty-liver models (86, 87). Other glucagon-like peptide-1 receptor agonists, sodium–glucose cotransporter 2 inhibitors, thiazolidinediones, and weight-loss therapies may indirectly reduce lipotoxic stress (88). Clinical availability of these agents does not mean that ferroptosis is an established mediator of their benefit. Across completed trials of repurposed ferroptosis-modulating drugs in other fields, ferroptosis-specific pharmacodynamic endpoints and prospective confirmation of ferroptosis as the therapeutic mechanism have generally been absent (89). T2DM translation should therefore begin with pathway-engagement studies and validated tissue-relevant endpoints rather than infer mechanism from glucose lowering alone.

### 7.5 Natural products, RNA regulation, and targeted delivery

Berberine, polydatin, silibinin, puerarin, plantamajoside, grape-seed procyanidins, and multicomponent traditional formulations regulate NRF2–GPX4, xCT, AKT1, AGE–RAGE, or mitophagy pathways in cellular or animal models. MicroRNAs and N6-methyladenosine-dependent regulation also provide candidate intervention points. These findings are useful for target discovery but should not be presented as evidence of clinical efficacy. Variable composition, uncertain exposure, batch effects, and bidirectional redox actions remain major obstacles. Mesenchymal-stromal-cell-derived exosomes and engineered nanocarriers can modulate ferroptosis-related pathways and may improve local delivery in preclinical disease models (90). For β-cell targeting, however, manufacturing consistency, cargo heterogeneity, biodistribution, immunogenicity, off-target uptake, and long-term safety remain unresolved; these platforms are experimental rather than clinically validated T2DM treatments. **Table 2** separates clinical availability from ferroptosis-specific evidence and the next evidentiary step.

**Table 2:** Clinical availability and ferroptosis-specific evidence of interventions along the lipotoxicity–ferroptosis axis.

Intervention/representative strategy	Intended metabolic or ferroptosis-directed action	Current clinical status	Ferroptosis-specific evidence in T2DM	Principal limitation and next evidentiary step
Weight loss and exercise	Reduce fatty-acid overflow, ectopic lipid, and oxidative demand	Routine obesity/T2DM management	Clinical metabolic benefit established; ferroptosis mediation unproven (56, 57, 81, 88)	Prospective pathway-engagement studies with tissue-interpretable endpoints
Metformin and GLP-1 receptor agonists	Metabolic unloading plus possible redox-defense reinforcement	Established glucose-lowering/weight-management therapies	Mainly cellular or animal ferroptosis evidence (20, 86, 87)	Do not infer mechanism from glycemic benefit; measure prospective pharmacodynamic endpoints
PPAR-directed therapies	Improve insulin sensitivity and lipid partitioning	Some agents are clinically used	Ferroptosis-specific T2DM evidence limited	Separate known metabolic effects from ferroptosis mechanism and assess tissue selectivity
DGAT1/lipid droplets, CD36, SCD1	Redirect lipid uptake, storage, or desaturation	Predominantly preclinical as ferroptosis/T2DM-progression targets	Mechanistic and model-specific	Risk of tissue-opposite effects; require cell-specific models
ACSL4/LPCAT3	Reduce oxidizable PUFA-phospholipid incorporation	Preclinical mechanistic target	Cellular/animal evidence; no validated human T2DM therapy	Define physiological lipid costs, dose, duration, and tissue targeting
System Xc−/GSH/GPX4 and NRF2	Detoxify phospholipid hydroperoxides	Ferroptosis-directed use remains preclinical	Most concentrated downstream experimental evidence	Long-term immune, metabolic, drug-interaction, and tumor-surveillance risks
FSP1/CoQ10, DHODH, GCH1/BH4	Increase GPX4-parallel ferroptosis resistance	Mechanistic candidates	Strong general ferroptosis biology; sparse direct T2DM evidence	Tissue-specific causality, exposure, and safety studies required
Iron chelation	Reduce redox-active iron	Approved for defined iron-overload disorders, not routine T2DM	Context-dependent metabolic/iron-overload model evidence (45)	Do not treat serum ferritin alone; establish tissue iron excess and avoid iron deficiency
TfR1, NCOA4, ferroportin modulation	Alter uptake, ferritinophagy, or export	Experimental/preclinical	Model-specific; no validated clinical T2DM application	Demonstrate cell selectivity and systemic iron safety
Exosomes/nanocarriers and β-cell-targeted delivery	Increase local exposure and reduce systemic effects	Experimental proof of concept	General ferroptosis and exosome evidence; direct T2DM validation limited (90)	Manufacturing, cargo heterogeneity, biodistribution, immunogenicity, and long-term safety

ACSL4, acyl-CoA synthetase long-chain family member 4; BH4, tetrahydrobiopterin; CD36, cluster of differentiation 36; CoQ10, coenzyme Q10; DGAT1, diacylglycerol O-acyltransferase 1; DHODH, dihydroorotate dehydrogenase; FSP1, ferroptosis suppressor protein 1; GCH1, GTP cyclohydrolase 1; GLP-1, glucagon-like peptide-1; GPX4, glutathione peroxidase 4; GSH, glutathione; LPCAT3, lysophosphatidylcholine acyltransferase 3; NCOA4, nuclear receptor coactivator 4; NRF2, nuclear factor erythroid 2-related factor 2; PPAR, peroxisome proliferator-activated receptor; SCD1, stearoyl-CoA desaturase 1; T2DM, type 2 diabetes mellitus; TfR1, transferrin receptor 1. Clinical translation remains limited: completed trials of repurposed ferroptosis-modulating agents in other fields generally lacked ferroptosis-specific pharmacodynamic endpoints or prospective confirmation of ferroptosis as the therapeutic mechanism (89).

Clinical availability does not imply that ferroptosis is an established therapeutic mechanism. No ferroptosis-directed intervention is currently validated or approved to prevent progression from insulin resistance to T2DM.

### 7.6 Timing, combination therapy, and cell selectivity

Intervention timing may determine whether ferroptosis modulation adds value. During compensation, reduction of energy surplus and lipid overflow addresses the dominant upstream process and may prevent ferroptosis sensitization without a dedicated inhibitor (81). During prediabetes or rapidly declining β-cell function, a transient therapy that reinforces lipid-peroxide defense could theoretically preserve functional mass while metabolic unloading takes effect (20, 58). In established T2DM with extensive dedifferentiation or cell loss, ferroptosis inhibition alone is unlikely to restore β-cell identity or reverse systemic insulin resistance.

Combination strategies should therefore be mechanistically complementary. A weight-loss or insulin-sensitizing treatment could reduce substrate and ROS generation, while a tissue-targeted intervention could stabilize vulnerable β cells (91). Blocking ACSL4-dependent remodeling and reducing labile iron might be synergistic but could also disrupt essential lipid and mitochondrial functions (92). Dose, duration, and tissue exposure must be optimized to lower pathological lipid peroxidation without abolishing physiological redox signaling (93). Importantly, cell-selective delivery is not merely a pharmacokinetic convenience: it may be necessary to protect β cells while permitting removal of pathogenic inflammatory cells. **Figure 3** maps these interventions by disease stage, translational maturity, and cell selectivity.

**Figure 3:**
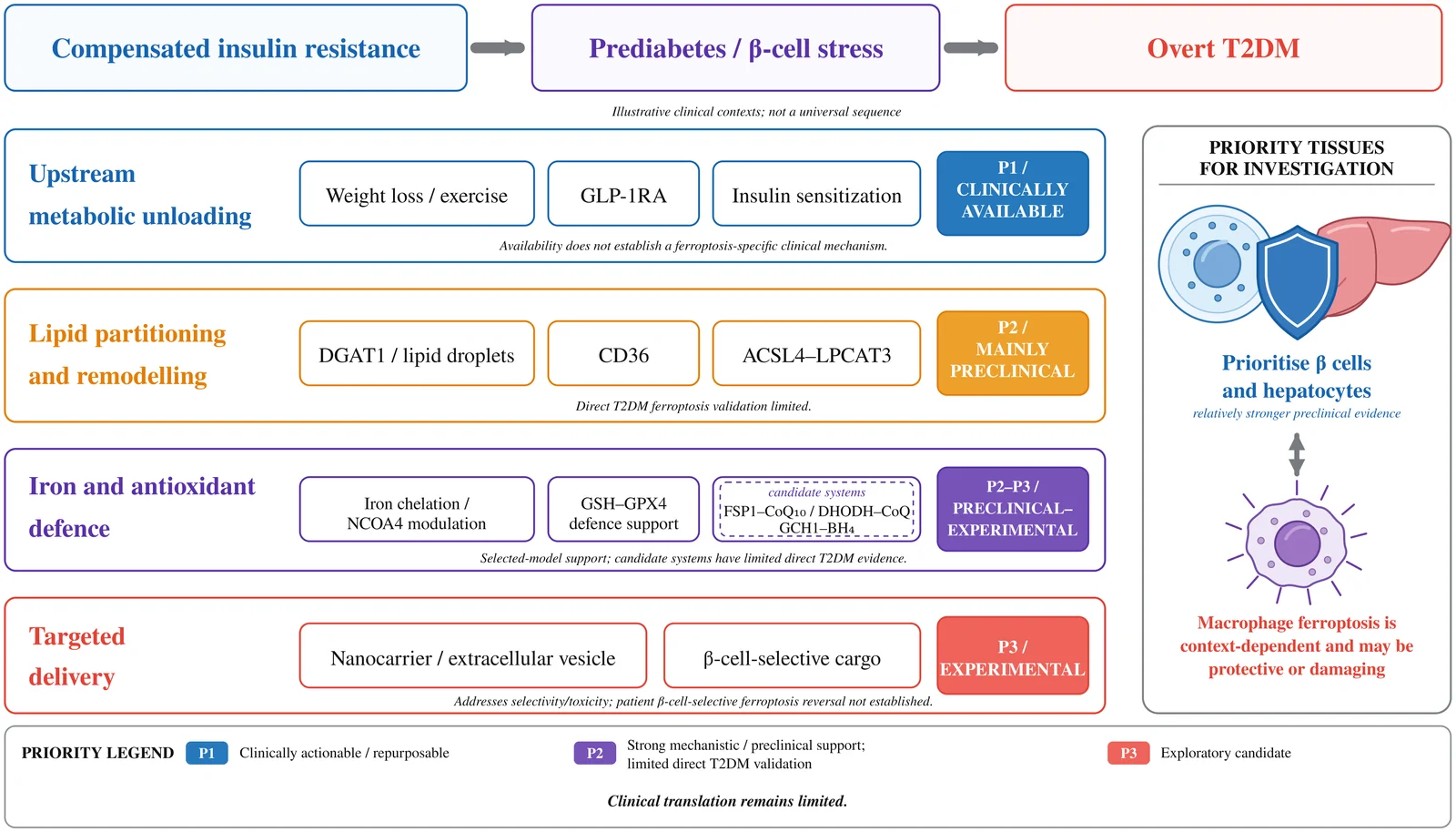
Evidence-stratified therapeutic map. P1 denotes clinically available or repurposable metabolic interventions whose ferroptosis mediation remains unproven; P2 denotes strategies with mechanistic or preclinical support but limited direct T2DM validation; and P3 denotes exploratory candidates. Established weight-loss and glucose-lowering interventions have clinical metabolic benefit, but ferroptosis is not a clinically proven mediator of that benefit. ACSL4/LPCAT3 modulation, GPX4-network reinforcement, FSP1, DHODH, GCH1–BH4, NCOA4 modulation, and targeted delivery remain preclinical or experimental. Iron chelators are clinically used for defined iron-overload disorders but are not established ferroptosis-directed treatments for routine T2DM. β cells and hepatocytes are prioritized for investigation because their preclinical evidence is relatively stronger, whereas macrophage effects remain context dependent. No ferroptosis-directed therapy is validated or approved to prevent progression from insulin resistance to human T2DM. Original conceptual schematic constructed and manually refined in Adobe Illustrator (Adobe Inc.) using vector-design and AI-assisted functions; no published image was reproduced or adapted.

## 8 Candidate biomarker framework and requirements for clinical validation

Clinical translation is constrained by the absence of a specific and validated biomarker of ferroptosis in T2DM. Circulating malondialdehyde, 4-hydroxynonenal, oxidized low-density lipoprotein, ferritin, GPX4, and total antioxidant capacity are influenced by inflammation, liver disease, diet, medication, renal function, and sample handling (94). They generally do not identify the tissue of origin. Magnetic-resonance iron or fat quantification can provide organ-level localization in liver or pancreas but cannot by itself identify the affected cell type or prove ferroptotic death (95). Tissue GPX4, SLC7A11, ACSL4, labile iron, or oxidized phospholipids gain spatial meaning only when measured in biopsy, single-cell, spatial-omics, or validated imaging contexts, and even then remain supportive rather than diagnostic readouts unless iron dependence and rescue are demonstrated. An exploratory multidimensional panel is more defensible than any single readout, but it is not currently a validated clinical classifier. A lipid-load domain could include non-esterified fatty acids, ceramide or diacylglycerol species, and ectopic liver or pancreatic fat (88). An iron domain could include ferritin, transferrin saturation, and organ-specific magnetic-resonance iron quantification, while recognizing that serum ferritin does not measure the intracellular labile iron pool. A lipid-peroxidation domain could include oxidized phospholipids, F2-isoprostanes, or 4-hydroxynonenal adducts (96). A functional domain could include insulin sensitivity, oral-glucose-tolerance-test-derived measures, C-peptide, and β-cell secretory indices (97, 98), which quantify metabolic function rather than the mechanism of cell death. Such panels should presently be used for hypothesis generation and prospective validation, not to diagnose a clinically established “ferroptosis-susceptible phenotype.” **Table 3** summarizes candidate domains and their tissue interpretability.

**Table 3:** Candidate biomarker domains, tissue interpretability, and validation gaps in T2DM-associated ferroptosis.

Biomarker domain/candidate	Specimen or measurement	Tissue interpretability	Major confounder	Current validation for T2DM ferroptosis
Ferritin and transferrin saturation	Blood	Systemic iron status; does not measure intracellular labile iron	Inflammation, liver disease, infection, anemia	Not diagnostic; supports iron-context assessment only
MRI iron quantification	Liver, pancreas, or other organ imaging	Organ-level, not cell-type or death-mechanism specific	Fibrosis, fat, scanner/sequence differences	Not validated to diagnose ferroptosis
Oxidized phospholipids, F2-isoprostanes, 4-HNE	Plasma, urine, or tissue	Usually non-specific in circulation; tissue localization requires biopsy/imaging	Diet, inflammation, sample handling, other oxidative-death pathways	Supportive lipid-peroxidation readouts only
GPX4, SLC7A11, ACSL4	Tissue, single-cell/spatial omics, or experimental imaging	Potentially cell localized when measured in tissue	Expression does not equal activity or cell death	Not diagnostic without iron dependence and rescue
NEFA, ceramides, DAG, liver/pancreas fat	Blood lipidomics or imaging	Lipotoxic burden; partly organ localized by imaging	Diet, medication, obesity phenotype	Not ferroptosis biomarkers
C-peptide and OGTT-derived indices	Blood-based functional testing	β-cell function, not death mechanism	Renal function, glucose exposure, medication	Clinical functional outcomes, not ferroptosis-specific

4-HNE, 4-hydroxynonenal; ACSL4, acyl-CoA synthetase long-chain family member 4; DAG, diacylglycerol; GPX4, glutathione peroxidase 4; MRI, magnetic resonance imaging; NEFA, non-esterified fatty acids; OGTT, oral glucose tolerance test; SLC7A11, solute carrier family 7 member 11; T2DM, type 2 diabetes mellitus.

None of these markers, alone or as a composite panel, is currently validated to diagnose ferroptosis or a clinically established “ferroptosis-susceptible phenotype” in T2DM.

Potential treatment windows should be investigated prospectively rather than assumed from current biomarkers. Compensated insulin resistance should continue to be managed primarily through established metabolic care, including weight loss and lipid unloading (88). Individuals with MASLD, tissue iron accumulation, or high lipid-peroxidation-associated signals could be prioritized for observational or short-term mechanistic studies, but these findings do not currently justify ferroptosis-directed treatment. Rapid β-cell functional decline may define a group in which GPX4-network or islet-targeted approaches warrant preclinical and early translational investigation. The immediate objective is to validate whether combinations of lipid burden, iron handling, lipid peroxidation, and β-cell function identify a reproducible biological subgroup and predict pathway engagement. No such panel is currently validated for routine clinical stratification.

## 9 Discussion: controversies and a translational research agenda

The first priority is rigorous causal identification. Future studies should combine oxidized-phospholipid lipidomics, labile iron measurements, ultrastructural analysis, genetic manipulation of GPX4 or ACSL4, and rescue with at least two mechanistically distinct ferroptosis inhibitors. Parallel inhibition of apoptosis, pyroptosis, and necroptosis is needed when several death pathways are plausible. Changes in ROS, malondialdehyde, GPX4, or iron in isolation should be described as compatible with ferroptotic stress, not definitive proof of ferroptosis (99).

The second priority is human relevance. Millimolar palmitate exposure and high-fat-diet/streptozotocin models may introduce acute toxicity or insulin deficiency that does not mirror human T2DM (100). Human primary islets, stem-cell-derived islets, adipose–liver–muscle–islet microphysiological systems, and chronic exposures that reproduce patient-level fluctuations are needed (101). Studies should report fatty-acid binding conditions, glucose concentration, exposure duration, sex, age, and tissue iron status, as each can alter the ferroptosis threshold.

The third priority is resolution of cell-type and disease-stage effects. Macrophage Gpx4 loss may improve obesity-associated insulin resistance, whereas protection of β-cell GPX4 generally preserves function (58). This is not necessarily contradictory: ferroptosis may remove harmful immune cells while damaging irreplaceable parenchymal cells. Cell-specific genetic models, spatial omics, lineage tracing, and repeated measurements across compensation, prediabetes, and overt T2DM are required to define when ferroptosis is adaptive, pathogenic, or merely a bystander.

The fourth priority is staged clinical mechanism testing. Longitudinal lipidomic, iron-metabolism, and β-cell-function analyses can first be embedded in weight-loss, metformin, or glucagon-like peptide-1 receptor agonist cohorts to test reproducibility, tissue interpretability, and association with outcomes. Candidate panels must then be prospectively validated before they are used for trial enrichment or treatment selection. Only after validation should proof-of-mechanism trials compare targeted pathway modulation plus standard care with standard care alone. Primary outcomes should include insulin sensitivity and C-peptide-based β-cell function; secondary mechanistic outcomes could include oxidized phospholipids and organ-specific iron imaging (102). Safety surveillance must include anemia, infection, liver injury, impaired exercise capacity, and potential effects on tumor surveillance. Clinical availability of a repurposed drug should never be taken as proof that ferroptosis is the mechanism of benefit (89).

The proposed axis has explanatory value because it integrates metabolism and cell fate, but it should not become a label applied to every observation of oxidative stress in diabetes. Its validity will depend on falsifiable predictions. For example, β-cell-specific reduction of oxidizable PUFA-phospholipids should delay decompensation without necessarily correcting upstream muscle insulin resistance; selective elimination of ferroptosis-prone inflammatory macrophages may improve insulin action, whereas systemic inhibition may not; and a composite high-lipid/high-iron/low-defense phenotype should predict response better than ferritin or GPX4 alone. Testing such predictions will determine whether the lipotoxicity–ferroptosis axis is a causal therapeutic framework or only a useful description of convergent stress.

## 10 Conclusion

Lipotoxicity can supply oxidizable membrane lipids, oxidative pressure, and progressive failure of antioxidant defense, thereby increasing ferroptosis susceptibility. A permissive redox-active iron pool remains a distinct, cell- and tissue-specific requirement. Ferroptosis may then convert reversible metabolic stress into persistent decline in β-cell function and cell number in selected experimental contexts. Current evidence is strongest in pancreatic β cells and liver, whereas direct causality in skeletal muscle and human tissues remains limited. Upstream lipid unloading has the greatest clinical maturity; GPX4–ACSL4 signaling, labile iron, parallel ferroptosis-defense systems, and targeted delivery are predominantly preclinical or candidate strategies. Translation will require rigorous ferroptosis definitions, cell- and stage-specific models, human-relevant experimental systems, and prospective validation of tissue-interpretable biomarker panels. Under these conditions, the lipotoxicity–ferroptosis axis may evolve from an explanatory model into a testable strategy for preventing progression from insulin resistance to T2DM.
